# The Impact of the Electronic Cigarettes Vaping on the Salivary Superoxide Dismutase, Zinc and Copper in Comparison to Tobacco Cigarette Smoking

**DOI:** 10.1155/ijod/5582470

**Published:** 2026-10-14

**Authors:** Shahba’a Munther

**Affiliations:** ^1^ College of Dentistry Department of Pediatric and Preventive Dentistry University of Baghdad Baghdad Iraq uobaghdad.edu.iq

**Keywords:** electronic cigarette using, salivary copper, salivary superoxide dismutase, salivary zinc, tobacco cigarette smoking

## Abstract

**Background:**

Electronic cigarettes (ECs) have adverse effects on human health similar to tobacco cigarettes. This cross‐sectional study was conducted to determine the impact of ECs versus tobacco cigarettes on salivary superoxide dismutase (SOD) and its cofactors, zinc and copper, as well as their effect on the periodontal health status.

**Methods:**

One hundred sixty‐five dental students (21–23 years) were categorised into: tobacco cigarette smokers, EC users and non‐smokers (control). Unstimulated saliva was collected to determine the concentrations of salivary SOD, zinc and copper were analysed. Periodontal status was assessed by periodontal disease index (PDI).

**Results:**

The salivary SOD, zinc and copper levels were slightly increased in non‐smokers, followed by EC users, while they were slightly decreased in tobacco cigarette smokers, reaching significant levels (*p* ≤ 0.001). The smoking status (including the smoking types) showed a statistically highly significant effect on the three investigated biochemical markers (*p* ≤ 0.001). The periodontal index scores were elevated in tobacco smokers compared with EC users, while they were reduced in non‐smokers; these differences reached significant levels (*p* ≤ 0.001). Negative correlations were recorded between the periodontal index mean levels and salivary SOD concentrations across the three groups (correlation coefficients = −0.441, −0.545 and −0.545), which reached highly significant levels (*p* ≤ 0.001). Periodontal index mean levels also showed significant negative correlations with zinc levels (−0.319, −0.323 and −0.343; *p* ≤ 0.05). The correlations between the periodontal index mean and copper concentrations were non‐significant.

**Conclusion:**

Zinc, copper and SOD levels recorded statistically significant differences among the three groups. However, the magnitude of such differences was very small. Therefore, although statistically significant, these findings may not provide clinically meaningful or biologically relevant changes, where they could be affected by the variability within the study group.

## 1. Introduction

Tobacco smoking has a great negative effect on human health, affecting almost every system in the human body. It is associated with different health risks, including cancers, cardiovascular and respiratory diseases, various chronic conditions, premature ageing, low bone mass, osteoporosis and several oral diseases [1].

To reduce the health hazards associated with traditional smoking, new devices were introduced that heat a solution composed of (nicotine, vegetable glycerine, propylene glycol, chemical additives and flavourings), producing an aerosol inhaled by the user [2–4]. Reports suggested that using these products, known as electronic cigarettes (ECs) or e‐cigarettes, may aid in tobacco smoking cessation; however, definitive data are lacking. ECs were not intended as therapeutic or pharmacological interventions.

The use of ECs increased, leading to population exposure to their aerosol ingredients [5, 6]. The liquids used in ECs are composed of a wide range of substances beyond nicotine, including acrolein, acetone, glycerol, ethanol, formaldehyde, 1, 3‐butadiene and other volatile organic compounds (FDI, 2021) [4]. The composition of these chemicals may differ from brand to brand and from one production batch to another. It is important to highlight that some of the toxic agents found in tobacco smoke, such as butanol, formaldehyde, and acetaldehyde, are also present in ECs [2, 3, 6, 7].

Reports and documents recorded the negative health effects of EC use, including cardiovascular, respiratory system, and nervous systems [8–10]. Oral health is also affected by ECs, with evidence that EC users have reported an elevated risk of dental decay, periodontal diseases, xerostomia, irritation of the oral mucosa, microbial dysbiosis, and oral cancer [1, 11, 12].

Both electronic and traditional cigarettes may inhibit the antioxidant defence system of saliva, resulting in poor oral health outcomes [9, 12]. Salivary superoxide dismutase (SOD) is one of these important antioxidant enzymes, which participates in the oral antioxidant defence system by reducing oxidative stress. The SOD is secreted in both saliva and gingival crevicular fluid, which requires both copper and zinc ions as essential cofactors for its proper function [13, 14]. However, there are limitations in the studies regarding this aspect. This work was designed to determine the consequence of ECs versus tobacco cigarettes on salivary SOD, zinc and copper and their relations to periodontal health.

## 2. Materials and Methods

The study population involved in this cross‐sectional study consisted of dental students from the Dental College in Baghdad University, whose ages ranged from 21 to 23 years. This could limit the generalizability of the findings, but it could provide a more homogeneous group. Informed consent was obtained, and each participant was asked to answer a designed questionnaire to determine their smoking status. The individuals who have reported that they are smokers were asked to answer a self‐administered questionnaire to report the type of smoking they use, whether it is traditional tobacco cigarettes or ECs, in addition to their relevant information. Questionnaires that did not meet the inclusion criteria were excluded; the final sample size was 165 individuals, divided into three groups of 55 students in each: tobacco cigarette smokers, EC users and non‐smokers (control). Data collection extended for 3 months, from April 2023 to July 2023.

Participants in the EC group reported that they are exclusive users of ECs on daily basis (approximately one tube per day) of the available electronic liquid in the market containing (20–35 mg/mL) salt nicotine for 2–3 years; they did not have a previous history of smoking traditional tobacco cigarettes or a history of passive exposure to indoor smoking [15]. In a similar manner, individuals of the tobacco cigarette group reported (5–15 cigarettes/day) for 2–3 years, and they did not have a history of EC use and passive exposure to indoor smoke [16]. Any dual‐uses were excluded from the study. All participants in both groups of smokers reported that they have no history of systemic diseases and that they are not taking any medications or nutritional supplements.

The periodontal health status was assessed in all three study groups using the periodontal disease index (PDI) introduced by Ramfjord [17], which included gingival, plaque and calculus components. This index was chosen to achieve the goal of the study, which focuses on the assessment of plaque accumulation, calculus deposition and gingival inflammation. Although newer indices are recommended for the diagnosis of periodontal health status, they require additional clinical and radiographic parameters that do not align with the scope of this study. Unstimulated saliva samples were collected according to the method described by Tenovuo and Lagerlöf [18].

Salivary SOD was measured using the Abcam ELISA Kit ab283972 (Cambridge, United Kingdom). The assay provides quantitative analysis of honestly significant difference (HSD) in biological samples, including saliva, serum, plasma, tissues and cell lysates. In this test, the microplates were pre‐coated with polyclonal antibodies specific for HSD. The biotinylated detection antibody and the streptavidin–peroxidase conjugate were applied to form the antibody–antigen complex and then the unbound components were washed out. The substrate solution was added, the reaction was stopped and finally the colour intensity was measured.

The salivary zinc level was determined using the Abnova Corporation Zinc Assay Kit (KA1619, Taiwan), which provides a quantitative assessment of zinc in biological fluids. Zinc in the sample reacted with a chromogen to form a specific coloured complex and then the complex absorbance was measured at 425 nm.

The salivary copper levels were measured using the atomic absorption spectrophotometry (Buck 210 VGP, USA).

The analysis of the studied biomarkers was performed according to standard laboratory procedures. Both intra‐ and inter‐assay variability were determined and recorded to ensure analytical reliability. Duplicate analysis of the samples was done to decrease the measurement error. For each biomarker, a calibration curve was constructed. Regarding the physiological and collection variability, the concentrations of proteins were normalised to total salivary protein levels. Furthermore, the salivary flow rate was reported and determined in the analysis where appropriate. All participants were instructed to stop smoking or vaping and eating for a standardised period before saliva collection. All of the mentioned procedures were applied to reduce pre‐analytical and analytical variability.

The sample size for this work was determined using G. Power software Version 3.1.9.7 (Franz Faul, Universität Kiel, Germany). The analysis was based on one‐way analysis of variance (ANOVA) with three groups. The significance level was 0.05, the effect size was 0.25 (medium) and the statistical power was 0.80. The required sample size was 159 participants. To overcome the potential missing data or exclusion of participants and to enhance the research findings, 165 participants were examined as a total sample size (55 participants in each group).

The Ethics Committee in the Dental College of Baghdad University approved this work, the approval code is 729322. The research purpose was explained to all students who agreed to participate using informed consent. The work conformed to ethical criteria in the Helsinki Declaration issued by the World Medical Association.

The data were analysed by applying the SPSS software (IBM Corp., Version 21). Before the statistical analysis, both the Shapiro–Wilk test and Levene’s test were applied to determine the normality and homogeneity of variances. Results showed that the data were aligned with the requirement for parametric analysis (*p* > 0.05). Therefore, multivariate ANOVA (MANOVA), if it showed statistically significant results, one‐way univariate ANOVA would be used. To determine the correlations among the variables, Pearson’s correlation coefficient was used. Post hoc analysis was applied using Tukey’s HSD test following the ANOVA test to evaluate the differences in significance among groups. A 95% confidence level was used (*p* ≤ 0.05). The Bonferroni correction was applied to avoid type 1 error using an adjusted alpha level of 0.0167 (0.05/3).

## 3. Results

A total number of 165 dental students were involved in this study, divided into three groups with 55 students in each according to smoking status and type. All the selected students completed saliva collection and assessment of periodontal health status, and no data were excluded.

Salivary SOD was slightly elevated in non‐smokers, followed by EC users, while it was slightly decreased in tobacco cigarette smokers, with the mean values of 1.527 ± 0.052 µg/dL, 1.497 ± 0.031 µg/dL and 1.474 ± 0.031 µg/dL, respectively. A similar pattern was noted for the salivary copper and zinc levels. The mean values of copper were 0.348 ± 0.005 µg/dL in non‐smokers, 0.345 ± 0.005 µg/dL in EC users and 0.342 ± 0.006 µg/dL in tobacco cigarette smokers. The mean values of zinc were 0.547 ± 0.005 µg/dL, 0.545 ± 0.002 µg/dL and 0.543 ± 0.003 µg/dL, respectively (Table 1). One‐way ANOVA indicated highly significant differences among the three groups for the three biochemical variables (salivary SOD, zinc and copper) (*p* ≤ 0.001). The significance was across all three groups as confirmed by the post hoc component. One‐way MANOVA showed a highly significant overall effect of smoking types on the salivary SOD, zinc and copper (*p* ≤ 0.001) (Table 2).

**Table 1:** Distribution of salivary superoxide dismutase, zinc and copper among the studied groups.

The variables		Superoxide dismutase (μg/dL)		Zinc (μg/dL)		Copper (μg/dL)	
The groups	No.	Mean	SD	Mean	SD	Mean	SD
Control	55	1.527	± 0.052	0.547	± 0.005	0.348	± 0.005
Electronic cigarette	55	1.497	± 0.031	0.545	± 0.002	0.345	± 0.005
Tobacco cigarette	55	1.474	± 0.031	0.543	± 0.003	0.342	± 0.006
Total	165	1.499	± 0.045	0.545	± 0.004	0.345	± 0.006

Abbreviations: dL, decilitre; μg, microgram; No, number; SD, standard deviation.

**Table 2:** Statistical differences and effects of smoking types and non‐smoking on the studied parameters.

Salivary parameters	One‐way ANOVA test			One‐way MANOVA test		
	*F*‐test		*p*‐Value	*F*‐test	*p*‐Value	Wilks’ lambda
Salivary superoxide dismutase	18.152^a^		≤0.001	6.973^a^	≤0.001	0.154
Zinc	9.860^a^		≤0.001			
Copper	11.985^a^		≤0.001			

Periodontal disease index	One‐way ANOVA test			One‐way MANOVA test		
	*F*‐test	*p*‐Value		*F*‐test	*p*‐Value	Wilks’ lambda
Periodontal component	12.463^a^	≤0.001		6.038^a^	≤0.001	0.136
Plaque component	17.154^a^	≤0.001				
Calculus component	13.448^a^	≤0.001				

^a^Highly significant.

Regarding the individual periodontal indices, the mean values of the gingival and periodontal components were higher in tobacco smokers (0.553 ± 0.397) than in EC users (0.407 ± 0.359). At the same time, they were lower in non‐smokers (0.200 ± 0.127), as shown in Table 3. The same patterns were recorded for the plaque component (0.522 ± 0.137, 0.447 ± 0.126 and 0.351 ± 0.128, respectively) and the calculus component (0.419 ± 0.125, 0.358 ± 0.142 and 0.271 ± 0.115, respectively) (Table 3). All of these differences were highly significant according to one‐way ANOVA (*p* ≤ 0.001). Post hoc component showed that each group was different from the other two groups for the three components at a significant level (*p* ≤ 0.001). One‐way MANOVA reported a highly significant overall effect of smoking types on the periodontal health status (*p* ≤ 0.001) as shown in Table 2.

**Table 3:** Means of the periodontal disease index components.

The components		Gingival and periodontal component		Plaque component		Calculus component	
The groups	No.	Mean	SD	Mean	SD	Mean	SD
Control	55	0.200	± 0.127	0.351	± 0,128	0.271	± 0.115
Electronic cigarette	55	0.407	± 0.359	0.447	± 0.126	0.358	± 0.142
Tobacco cigarette	55	0.553	± 0.397	0.522	± 0.137	0.419	± 0.125
Total	165	0.387	± 0.347	0.440	± 0.147	0.349	± 0.141

Abbreviations: No, number; SD, standard deviation.

The periodontal index means showed negative correlations with salivary SOD levels across the three groups (correlation coefficients = −0.441, −0.545 and −0.545), all of which were statistically significant (*p* ≤ 0.001). Periodontal index mean values recorded significant negative correlations with zinc (−0.319, −0.323 and −0.343; *p* ≤ 0.05) (Table 4). However, the correlations recorded between the mean values of the periodontal index and copper were not significant.

**Table 4:** Correlations between the periodontal index component and the measured salivary parameters.

Salivary parameters	Periodontitis					
	Non‐smokers (control)		Electronic cigarette smokers		Tobacco cigarette smokers	
	*r*	*p*‐Value	*r*	*p*‐Value	*r*	*p*‐Value
Superoxide dismutase	−0.441^a^	0.004	−0.545^a^	≤0.001	−0.545^a^	≤0.001
Zinc	−0.319^b^	0.045	−0.323^b^	0.042	−0.343^b^	0.030
Copper	0.011	0.945	−0.107	0.511	−0.262	0.102

*Note: r*, correlation coefficient.

^a^Highly significant.

^b^Significant.

Table 5 explored the relationship between salivary SOD and its cofactors (zinc and copper). The results indicated that salivary SOD was positively correlated with zinc (0.747, 0.346 and 0.506) and copper (0.316, 0.161 and 0.486); some of these correlations were significant (*p* ≤ 0.05), while others were highly significant (*p* ≤ 0.001).

**Table 5:** The correlations between the measured salivary parameters.

Superoxide dismutase						
Groups	Non‐smokers (control)		Electronic cigarette smokers		Tobacco cigarette smokers	
Parameters	*r*	*p*‐Value	*r*	*p*‐Value	*r*	*p*‐Value
Zinc	0.747^a^	≤ 0.001	0.346^b^	0.029	0.506^a^	≤ 0.001
Copper	0.316^b^	0.047	0.161	0.322	0.486^a^	≤ 0.001

*Note: r*, correlation coefficient.

^a^Highly significant.

^b^Significant.

## 4. Discussion

Findings of the present study support the previous data suggesting that EC use is associated with a harmful effect on human health, but to a lesser extent in comparison to tobacco cigarettes [19]. This investigation was aimed at assessing the consequences of ECs versus tobacco cigarettes on the periodontal health and salivary levels of SOD, zinc and copper.

Unstimulated saliva samples were collected according to the method described by Tenovuo and Lagerlöf [18]. Unstimulated saliva reflects the baseline physiological concentrations of antioxidants, vitamins and trace elements that improve the accuracy of measured biomarkers and decrease the dilution effects caused by stimulated saliva.

Salivary SOD, zinc and copper recorded statistically significant differences among the groups. However, the definite differences among the groups were small, which may be related to lower variability rather than a large biological effect. These results align with those of Ye et al. [20], Kamal and Shams [16] and Zięba [21], who reported that both tobacco and EC use enhance oxidative stress and deteriorate antioxidant defences in saliva, with tobacco smoking having a relatively more negative impact.

The nicotine and other harmful compounds in both types of smoking might lower the oral immune defences; in addition, they increase oxidative stress by an increase in free radical production. On the other hand, both smoking types were well known to reduce salivary flow, which will further decrease the antioxidant capacity and mineral content [22–25]. This mechanism may account for the relative decreased levels of salivary SOD, zinc and copper recorded in this study. The results of one‐way MANOVA supported these findings by recording a statistically significant effect of smoking status on all three salivary parameters.

In contrast to these results, there are some reports in the literature, such as the findings recorded by Baharavand et al. [26] and Jenifer et al. [27], which reflected an elevation in the salivary SOD in smokers; this could be a result of the compensatory antioxidant reaction in response to oxidative stress. Likewise, an increase in salivary antioxidant capacity with no significant difference between EC and tobacco smoking was recorded by Cichońska et al. [23].

Such contradictions regarding salivary SOD levels in smokers may be due to differences in duration and intensity of smoking and the stage of oxidative stress exposure. In the early stages of smoking, antioxidant enzymes, including salivary SOD, may elevate as a part of compensatory defence mechanism against reactive oxygen species (ROS). By continuous exposure, chronic oxidative stress may exceed antioxidant defences, resulting in depletion or reduction in enzymatic activity. The relatively short duration of smoking reported in this study (2–3 years) may reflect a traditional stage in which the antioxidant response begins to decrease as a result of chronic oxidative effect, rather than reflecting sustained compensatory elevation. In addition to that, variation in nutrition pattern, genetic antioxidant capability, sample size and differences in methodology may increase contradictions across researches [6, 27].

Regarding periodontal health, the mean scores of periodontitis, plaque and calculus were elevated in tobacco smokers than in EC users at a significant level, and they were lower in non‐smokers. These results aligned with the reports of Javed et al. [15], BinShabaib et al. [11] and Jeong et al. [28], which recorded the poorest periodontal status in tobacco smokers, moderate periodontal impairment in EC users and the best outcomes in non‐smokers. Similarly, Munther [29] and Abdul‐Jabbar and Hoobi [12] reported that both tobacco and EC have negative effects on the periodontal health status.

However, the prevalence of recorded significant periodontitis among the students was expected to be low. This could be due to their young ages. The observed periodontal differences may reflect the differences in plaque, calculus and gingival inflammation rather than advanced periodontal diseases.

There are several mechanisms which may explain these findings. Smoking has been reported to alter the oral microbiome and reduce salivary flow, which facilitates plaque accumulation, leading to calculus formation and contributing to periodontal disease [12]. In addition, salivary SOD performs a crucial role in the neutralisation of ROS such as superoxide anions (O_2_^−^), thus protecting the periodontal tissues and gingival fibroblasts from periodontal pathogens, in addition to its anti‐inflammatory effect. All these are important considerations of its role in the periodontal defence [13, 14]. Therefore, reduced levels of salivary SOD that are associated with smoking may facilitate the inflammation and disease progression in conjunction with calculus deposition.

Although statistically significant negative correlations were recorded between periodontal parameters and the studied variables, most of them were weak or moderate. These correlations propose that variables may tend to vary inversely. However, they interpret a relatively small portion of the variance and cannot be determined as strong evidence of an underlying mechanism. Further studies are recommended to support the mechanistic conclusions.

Salivary SOD enzymes have been classified into different types based on the metal cofactors and the folding of their proteins. The Cu/Zn‐SOD family, which binds both copper and zinc, is the main and first discovered form of the enzyme in humans. The enzymatic activity of salivary SOD also depends on the availability of these two metals [13, 14]. According to this fact, the positive correlations between salivary SOD and zinc and copper, as well as the negative correlations between zinc and periodontitis, supported the biological plausibility of these associations.

Although salivary SOD reported positive correlations with both zinc and copper, only zinc showed statistically significant negative correlations with CPI scores, while correlations with copper were non‐significant. There is a tight homeostatic regulation of copper that maintains its level in saliva relatively stable and limits its response to periodontal inflammation. On the other hand, zinc plays a significant role in immune response, tissue repair and inflammatory response, rendering zinc a more sensitive marker of periodontal health status. Furthermore, zinc is responsible for the structural stability of Cu/Zn‐SOD, while copper supports the catalytic function of the enzyme [30, 31].

The differences in the compositions of traditional and ECs might contribute to the differences in the severity of their effects. However, both smoking methods have a negative impact on general and oral health.

This work was limited by self‐reported smoking status, and the exposure intensity was not quantified using objective biomarkers or detailed measures such as pack‐years, vaping frequency, or puff counts. These factors may affect the results of the study. In addition to that, the sample was drawn from the student population in the College of Dentistry. These individuals may be better aware of smoking‐related risks than other populations in the country, and this might affect both their behavioural and biological variables. Other confounding factors, such as oral hygiene habits, brushing frequency, diet, alcohol use, stress, sleep, BMI, socioeconomic status, periodontal treatment history, were not considered in this work, which may affect the results. The absence of existing research dealing with both smoking types and their effect on salivary SOD and its cofactors also limits this work in terms of comparative analysis.

Although the absolute differences among the three groups were small and may not be translated into clinical differences, the statistical significance recorded among the three groups may give an indication that both traditional and ECs are associated with adverse effects on oral health, with traditional cigarettes being slightly more harmful. The greater impact determined with traditional cigarettes use may be explained by the higher concentration of toxic and carcinogenic substances found in these cigarettes compared to ECs. These findings showed that both types of smoking should be avoided and replaced by other lifestyle factors, such as a healthy diet, sports and oral hygiene practices.

Although this study showed that both types of smoking are harmful to oral health, with traditional cigarettes showing a great effect, the generalizability of these results may be limited. The study sample was restricted to dental students aged (21–23) collected from the Dental College at Baghdad University. Other patterns of use, oral hygiene practice and socioeconomic status may affect the degree of harm.

Further studies should aim to quantify the influence of tobacco and EC on SOD, zinc and copper levels in saliva. Larger and more diverse populations, in addition to other variables such as nutritional habits and socioeconomic status, should be considered in future works. Awareness regarding the adverse effects of all forms of smoking, including vaping, should be intensified.

## 5. Conclusion

Salivary zinc, copper and SOD levels recorded statistically significant differences among the three groups. However, the magnitude of such differences was very small. Therefore, although statistically significant, these findings may not provide clinically meaningful or biologically relevant changes, where they could be affected by the variability within the study group.

## Funding

There was no funding received for this work.

## Conflicts of Interest

The author declares no conflicts of interest.

## Data Availability

The data that support the findings of this study are available upon request from the corresponding author. The data are not publicly available due to privacy or ethical restrictions.
